# 20,000

**Published:** 1917-11

**Authors:** 


A Monthly Review of Medicine and Surgery

EDITOR AND PUBLISHER, DR. A. L. BENEDICT, 228 Summer St., corner of Elmwood Ave., Buffalo, (Address for all communications. Please make personal and telephone calls before 1 P. M.)

Third, in age, on the western continent. Independent, but supports professional organizations. News limited mainly to Western N. Y. and Penn.

Subscription $2.00 a year; $3.00 for two years in advance. Changes and errors in address or failure or duplication of delivery, should be reported immediately.

Yearly Volume 73 NOVEMBER, 1917 Number 4

### 20,000.

Do we realize the full significance of the estimate of physicians demanded for the present war, by our country? It means, at least, an eighth of the total number of men and women holding medical degrees; about a sixth of the men actually in medical practice. Allowing for the wise decision not to deprive the rural districts of physicians, it means that nearly a third of the male practitioners of cities are required. If the same age limits were applied to the medical department as to the general draft, it would take practically every physician under 31. Indeed, when we consider that few persons have been able to graduate in medicine in the last ten years, under 22, that the average age at graduation is 26, and also that even in the first 6-9 years after graduation, there is a not inconsiderable ratio of deaths, it is doubtful whether there are 20,000 male physicians in the country, under 31. Compare the requirement with the liberal allowance that must be made for physical disability and with the requirement, for men outside the medical profession, of only about 1 out of 5, for military purposes.

In mere numbers, this quota of medical men is impressive. With a very few exceptions, it is greater than any entire army in what is now the U. S., up to the War of 1812. With probably no exception, it exceeds any single combatant force of the Colonial Wars or that of the Revolution. It is the equivalent of a division up to the present, greater than a division in some of the present belligerent armies even at full strength, it amounts to 2/3 of the present theoretic division of the U. S. Army. Expressed in lower terms, it is equal to 6 full war strength regiments of the present, to 20 regiments as the term has usually been employed in the past, to 30 or 40 of the regiments that we have seen in parades or processions. It is equal to the total force required to maintain 4 or 5 miles of front in trenches. The Surgeon General of the U. S. Army will have under him, at the present estimate, a force of officers and enlisted men greater than the entire U. S. Army on a peace establishment.

Have we considered why an estimate of 20,000 medical officers has been made, when all present talk has been of an army of at most 2 million? Such an army would need about 10,000 medical officers according to past standards; 12,000 pro rata to even last year's statistics, when the number of all officers was relatively high, to allow for expansion to what was formerly the official war strength; 14,000 according even to the highest estimates up to the present war. The mere figures should bring home to us that the medical department is not a fixed quantity, that it is not merely liable to diminish according to ordinary laws of mortality so that its replenishment can be left to the future. There has been considerable loss by death and permanent disability, of the surgeons of other armies and our country will undoubtedly be called upon immediately to make good this loss on their part. But we cannot be blind to what the allowance beyond the needs of the immediate future signifies to us.

